# COVID-19 and the eye: Systemic and laboratory risk factors for retinopathy and detection of tear film SARS-CoV-2 RNA with a triplex RT-PCR assay

**DOI:** 10.1371/journal.pone.0277301

**Published:** 2022-11-09

**Authors:** Jessica G. Shantha, Tolulope Fashina, Victoria Stittleburg, Casey Randleman, Laura Ward, Matt Regueiro, David Krakow, Susanne L. Linderman, Carolyn Drews-Botsch, Rafi Ahmed, Jesse Waggoner, Steven Yeh

**Affiliations:** 1 Francis I. Proctor Foundation for Ophthalmic Research, University of California, San Francisco, San Francisco, CA, United States of America; 2 Truhlsen Eye Institute, Department of Ophthalmology, University of Nebraska Medical Center, Omaha, NE, United States of America; 3 Emory University School of Medicine, Atlanta, GA, United States of America; 4 Rollins School of Public Health, Emory University, Atlanta, GA, United States of America; 5 Emory Vaccine Center, Emory University, Atlanta, GA, United States of America; 6 Department of Global and Community Health, George Mason University, Fairfax, VA, United States of America; 7 Global Center for Health Security, University of Nebraska Medical Center, Omaha, NE, United States of America; Tsukazaki Hospital, JAPAN

## Abstract

**Purpose:**

To assess hospitalized COVID-19 inpatients for the prevalence of retinopathy and tear film SARS-CoV-2 RNA, and associated risk factors for their detection.

**Methods:**

Hospitalized COVID-19 patients underwent dilated ophthalmic examination and fundus photography. Conjunctival swabs were assessed for SARS-CoV-2 RT-PCR via a triple target assay. We assessed the relationships of retinopathy with clinical outcomes, systemic risk factors and laboratory data.

**Results:**

The median age was 59.5 years and 29 (48%) were female. Retinopathy associated with COVID-19 was observed in 12 of 60 patients (20%). The median age of patients with COVID-19 retinopathy was 51.5 compared to 62.5 years in individuals without retinopathy (p = 0.01). Median BMI was 34.3 in patients with retinopathy versus 30.9 in those without retinopathy (p = 0.04). Fifteen of 60 patients (25%) tested SARS-CoV-2 RNA-positive in their tear film without a relationship with timing of illness and hospitalization. The N2 gene was particularly sensitive with 18 of 19 eyes (94.7%) showing N2-positivity, including 2 patients with alpha variant-positivity (B.1.1.7).

**Conclusion:**

Retinopathy was observed in 20% of patients hospitalized for COVID-19. Patients with retinopathy were more likely to be younger and have higher BMI than hospitalized patients without retinopathy. Tear film SARS-CoV-2 RNA was detected in 25% of patients. The relationship of obesity and age with retinopathy requires further investigation.

## Introduction

The severe acute respiratory syndrome coronavirus 2 (SARS-CoV-2), leading to the ongoing coronavirus disease (COVID-19) pandemic, has resulted in over 517 million cases and 6.2 million deaths worldwide [1]. While SARS-CoV-2 infection may be asymptomatic or lead to severe respiratory illness with multi-organ involvement, ophthalmic manifestations have also been increasingly reported [2–4]. The reported prevalence of ophthalmic manifestations ranges from 1–32%, and these manifestations include both anterior segment [5–8] and retinal findings [9–17]. The reported retinal manifestations include retinal hemorrhage and cotton wool spots. Less commonly, retinal artery and venous occlusion may impact visual acuity [4, 9–17]. Recently, retinopathy in a cohort of severe COVID-19 inpatients was associated with the laboratory markers of coagulopathy and inflammation, suggesting thrombotic microangiopathy might play a role in the pathogenesis of retinal findings in COVID-19 [4].

In addition to ophthalmic manifestations associated with COVID-19, the precise role of the tear film as a transmission route either for pathogen entry or mucosal person-to-person transmission via hand-eye contact is unclear. Studies utilizing conjunctival swabs and Schirmer’s strips suggest that the prevalence of SARS-CoV-2 detection in tears and conjunctival secretion through RT-PCR ranges from 0 to 57.1%, and may vary with the presence of conjunctivitis and viral RNA load of the nasopharyngeal mucosa [18–24]. The potential for viral transmission through the tears remains a risk and the current personal protective equipment guidelines (PPE) recommend the use of goggles to healthcare professionals [25]. Other viruses known to infect ocular surface cells include well-known respiratory viruses such as adenovirus [26] and influenza virus, as well as emerging infectious diseases including SARS-CoV-1 and Zika virus [27]. SARS-CoV-1 and SARS-CoV-2 virus infect cells via the ACE-2 receptor and transmemb rane protease serine type 2 receptor, which have been identified in ocular surface epithelia, potentially serving as a conduit for viral transmission [28, 29].

We sought to prospectively assess the prevalence of retinal manifestations associated with COVID-19 inpatients and their relationship to systemic disease severity and laboratory indices within a university-based, tertiary referral care setting. We also sought to systematically assess SARS-CoV-2 viral RNA in the tear film and its relationship to ocular findings, host, and disease characteristics.

## Methods

This prospective observational, cross-sectional study was conducted at Emory University Hospital between January 1, 2021, and June 1, 2021. Hospitalized COVID-19 patients were offered study inclusion for ophthalmic examination and tear film collection for RT-PCR analysis. The study protocol was approved by the research ethics committee of Emory University (IRB ID STUDY00001071), and in accordance with the Declaration of Helsinki.

Inclusion criteria included adult patients with a confirmed diagnosis of COVID-19 through real-time RT-PCR assay of respiratory specimens, and who were admitted to the hospital ward or Intensive Care Unit (ICU) setting. The COVID-19 ophthalmology research team approached Hospital Medicine and ICU attendings about patients who would potentially be amenable for consideration of a research study, after which the study was discussed with the patient and/or appropriate family member with decision-making capacity. Informed consent was obtained from patients in the study or their legally authorized representatives for patients who were incapable of providing informed consent (e.g., intubation and sedation in an intensive care unit setting). Verbal consent was obtained with documentation via an electronic informed consent form consistent with COVID-19 transmission precautions and Emory University guidance. All patients or authorized representatives were given a copy of the informed consent for their review, additional questions, and discussion.

### Examination and sample collection

Ophthalmic exams were performed at bedside included anterior segment exam by penlight, and a dilated funduscopic exam with indirect ophthalmoscopy. Fundus photographs were taken with a portable fundus camera, Nidek DS-20 [Nidek, Inc, San Jose, CA]. *COVID-19 Retinopathy* was defined as any one of the following: 1) Retinal hemorrhage, 2) Cotton wool spots, 3) Retinal vein or artery occlusion, *and* no prior history of diabetic or hypertensive retinopathy. All exams and conjunctival swab samples were obtained in full personal protective equipment (PPE) for protection against respiratory transmission of SARS-CoV-2. PPE included a fluid-impervious gown, gloves, face shield, and N95 or KN95 mask.

For tear sample collection, a drop of 1% proparacaine was administered 1 minute before sample collection. A conjunctival swab was used to sweep the inferior conjunctival fornix of both eyes with a sterile sampling swab. The tip of the swab was placed in the sample tube containing EMAG lysis buffer solution (bioMérieux, Durham, NC). Samples collected at the time of ophthalmic exam were transported to the laboratory for analysis. Conjunctival swab samples were refrigerated in a 4°C refrigerator for batched RNA analysis.

### Data collection

Deidentified clinical information for enrolled patients were collected onto a secure data collection platform (DF/Net Research, Seattle, Washington, USA) using an iPad or computer. Prior to each patient visit, demographic characteristics, past medical history, and results of serological tests of each patient was extracted from the electronic medical record. Demographic information included age, sex, race, and ethnicity. Pertinent past medical history, ocular history and ophthalmic medications were documented. Hospital documentation and patients were specifically queried for a history of diabetic or hypertensive retinopathy prior to admission.

Medical data related to the patient’s hospitalization included ICU vs. ward admission, days of COVID-19 symptoms, respiratory or systemic symptoms, and hospitalization days. Interventions documented included mechanical ventilation, extracorporeal membrane oxygenation (ECMO), history of anticoagulation and corticosteroid use. Laboratory parameters documented included peak D-dimer, fibrinogen nadir, C-reactive protein, interleukin-6, platelets, hematocrit, and lymphocyte counts.

COVID-19 disease severity was classified according to the NIH COVID-19 Severity Scale (https://www.covid19treatmentguidelines.nih.gov/overview/clinical-spectrum/). Strata for COVID-19 severity included 1) Asymptomatic or presymptomatic infection; 2) Mild illness; 3) Moderate illness; 4) Severe illness; and 5) Critical illness. Asymptomatic or presymptomatic infections included individuals who tested positive for SARS-CoV-2 but did not have symptoms consistent with COVID-19 (e.g., hospitalization for appendicitis).

### RT-PCR

Real-time reverse transcription polymerase chain reaction (rRT-PCR) testing was performed for targets in the SARS-CoV-2 nucleoprotein and envelope E genes as well as RNase P [30]. A cycling threshold (Ct) of 40 was used as a cutoff for a positive result for N2, E, and RNase P genes. Conjunctival swab samples were considered RT-PCR positive if RNase P was detected and either N2 or E targets were positive (i.e., Ct < 40). Variant testing was performed using a laboratory-developed rRT-PCR targeting specific *spike* mutations, as previously described [31].

### Statistical analysis

Descriptive and inferential statistics were performed using R and Stata software. Demographic data including age, sex, race and ethnicity are summarized as means and standard deviations (SD) or frequencies with percentages as appropriate. The cohort was stratified into patients with and without COVID-19 retinopathy for analysis.

Patient demographic information, NIH COVID-19 severity score, baseline comorbidities, laboratory data and interventions were assessed as l risk factors for COVID-19 retinopathy with univariate and multivariate logistic regression. Unadjusted and adjusted odds ratios and 95% confidence intervals were calculated for each potential patient, disease, or laboratory factors.

Lastly, we assessed for relationships between tear film SARS-CoV-2 RT-PCR positivity, evidence of COVID-19 retinopathy, and systemic disease characteristics including duration of COVID-19 illness/symptoms, duration of hospitalization and NIH COVID-19 severity score. Two-sided, unpaired T-tests were utilized for comparison of continuous variables and Fisher’s exact test were utilized for proportions. A p-value of 0.05 was considered statistically significant.

## Results

Sixty patients were enrolled into this study from Emory University Hospital. The median age and interquartile range (Q1, Q3) of patients was 59.5 years (47, 69.5). Twenty-nine patients (48%) were female and 31 (52%) were male. The majority of patients were black (43, 72%). Other enrollees were white (11, 18%), Asian (1, 2%) or documented as unknown (5, 8%). Four patients were Hispanic ethnicity (7%) and all others (n = 56) were non-Hispanic (93%). Patients were stratified by the NIH COVID-19 severity scale for analyses. The majority of patients were deemed *critical* (n = 45, 75%) but other categories included *severe* (n = 2, 3%), *moderate* (n = 7, 12%), *mild* (n = 1, 2%) and *asymptomatic* (n = 5, 8%). Demographic and clinical features are summarized in Table 1.

**Table 1:** Demographic and baseline characteristics.

Patient Characteristics	All (n = 60 patients)	COVID-19 Retinopathy (n = 12 patients)	No COVID-19 Retinopathy (n = 48 patients)	Odds Ratio (95% CI)	p
**Age, Median Years (Q1, Q3)**	59.5 (47, 69.5)	51.5 (41, 56)	62.5 (48.5, 72.5)	0.75 (0.58, 0.95)	0.01^b^
**Sex**					
Female	29 (48%)	6 (50%)	23 (48%)	1.09 (0.31, 3.85)	0.90
Male	31 (52%)	6 (50%)	25 (52%)		
**Race**					
Black	43 (72%)	10 (83%)	33 (69%)	2.27 (0.44, 11.67)	0.18
White	11 (18%)	0 (0%)	11 (23%)		
Asian	1 (2%)	0 (0%)	1 (2%)		
Unknown	5 (8%)	2 (17%)	3 (6%)		
**Hispanic Ethnicity**	4 (7%)	1 (8%)	3 (7%)	1.30 (0.12, 13.78)	1.00
**NIH COVID-19 severity**					
Asymptomatic	5 (8%)	0 (0%)	5 (10%)		
Mild	1 (2%)	0 (0%)	1 (2%)		
Moderate	7 (12%)	1 (8%)	6 (13%)		
Severe	2 (3%)	0 (0%)	2 (4%)		
Critical	45 (75%)	11 (92%)	34 (71%)	4.53 (0.53, 38.48)^a^	0.19
**BMI, Median (Q1, Q3)**	32.9 (26.4, 36.5)	34.3 (33.3, 42.8)	30.9 (26.0, 36.5)	1.42 (1.01, 2.00)	0.04^b^
**ICU admission**	21 (36%)	6 (50%)	15 (32%)	2.13 (0.59, 7.73)	0.32
**Days of COVID illness, Median (Q1, Q3)**	6.5 (3, 12.5)	11.0 (4.5, 17.5)	6 (2.5, 10.5)	1.04 (0.99, 1.11)	0.16
**Days of hospitalization, Median (Q1, Q3)**	0 (0, 3.5)	3.5 (0, 9)	0 (0, 1.5)	1.04 (0.98, 1.12)	0.16
**Symptoms**					
Shortness of breath	44 (73%)	10 (83%)	34 (71%)	2.06 (0.40, 10.62)	0.48
Cough	31 (52%)	7 (58%)	24 (50%)	1.40 (0.39, 5.03)	0.75
Fatigue	24 (40%)	4 (33%)	20 (43%)	0.68 (0.18, 2.56)	0.74
Fever	16 (27%)	4 (33%)	12 (25%)	1.50 (0.38, 5.89)	0.72
Diarrhea	16 (27%)	5 (42%)	11 (23%)	2.40 (0.64, 9.09)	0.27
**Baseline comorbidities**					
Hypertension	40 (67%)	9 (75%)	31 (65%)	1.65 (0.39, 6.90)	0.74
Diabetes	24 (40%)	7 (58%)	17 (35%)	2.56 (0.70, 9.28)	0.19
Asthma	6 (10%)	2 (17%)	4 (9%)	2.15 (0.34, 13.42)	0.59
COPD	7 (12%)	0 (0%)	7 (15%)	1.68 (0.28, 9.95)	0.33
Obesity (BMI = >30)	34 (57%)	10 (83%)	24 (52%)	9.2 (1.1, 77.6)	0.04
Other immunosuppression	11 (18%)	1 (8%)	10 (21%)	0.35 (0.04, 3.00)	0.43

^a^Critical vs. non-critical

^b^Statistically significant, p<0.05

### COVID-19 retinopathy and ocular findings

**Fig 1:**
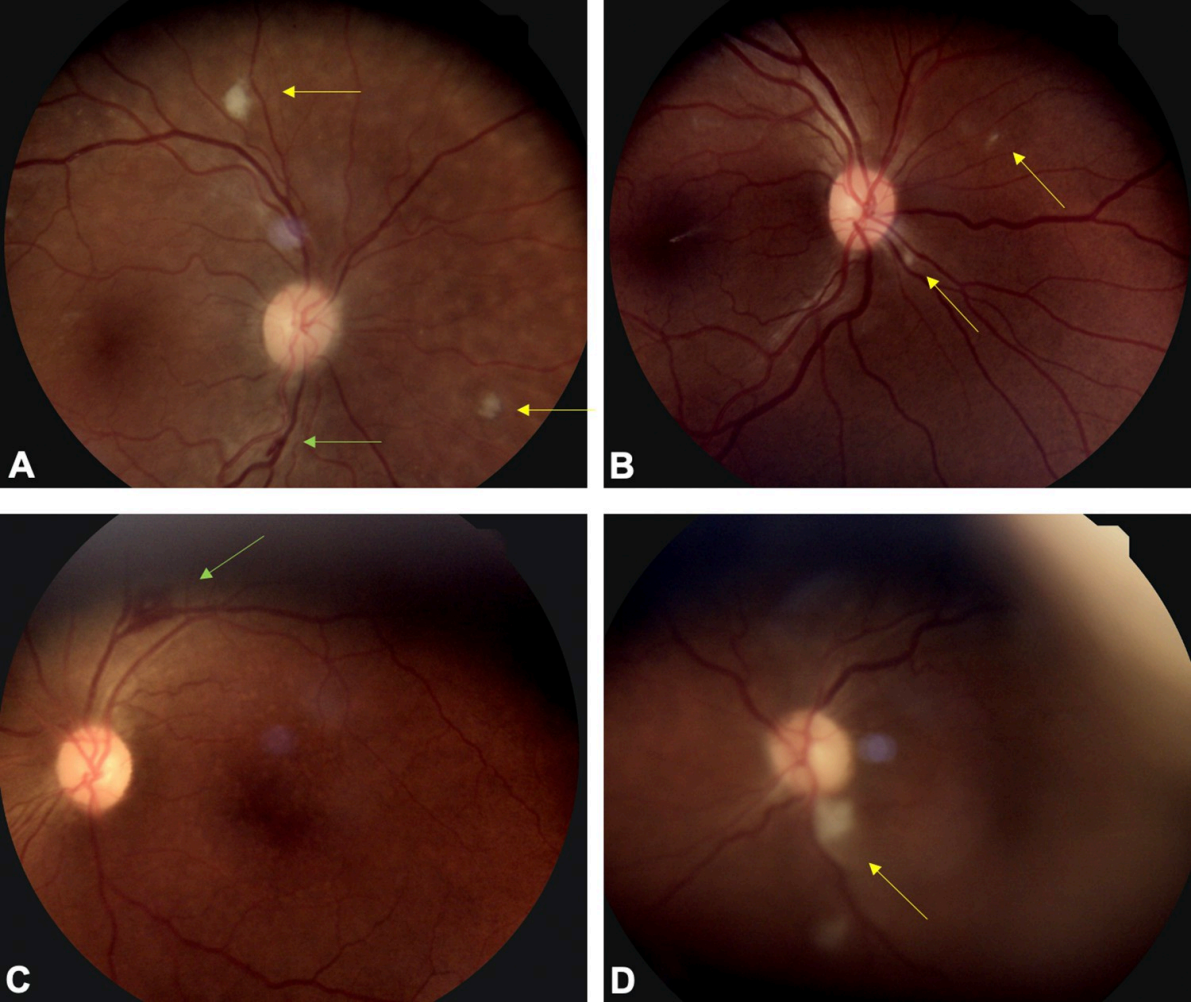
Fundus photographs of patients with COVID-19 associated retinopathy. (A) Fundus photo of peripapillary area and posterior pole shows cotton wool spots (yellow arrows) and linear nerve fiber layer hemorrhage (green arrow). (B) Fundus photo shows multiple cotton wool spots in peripapillary distribution (yellow arrows). (C) Fundus photo shows an approximately 750-micron nerve fiber layer hemorrhage in perivascular location (green arrow). (D) A fundus photo shows vascular tortuosity, attenuated arteries, and a large cotton wool spot inferior to the optic nerve (yellow arrow).

**Fig 2:**
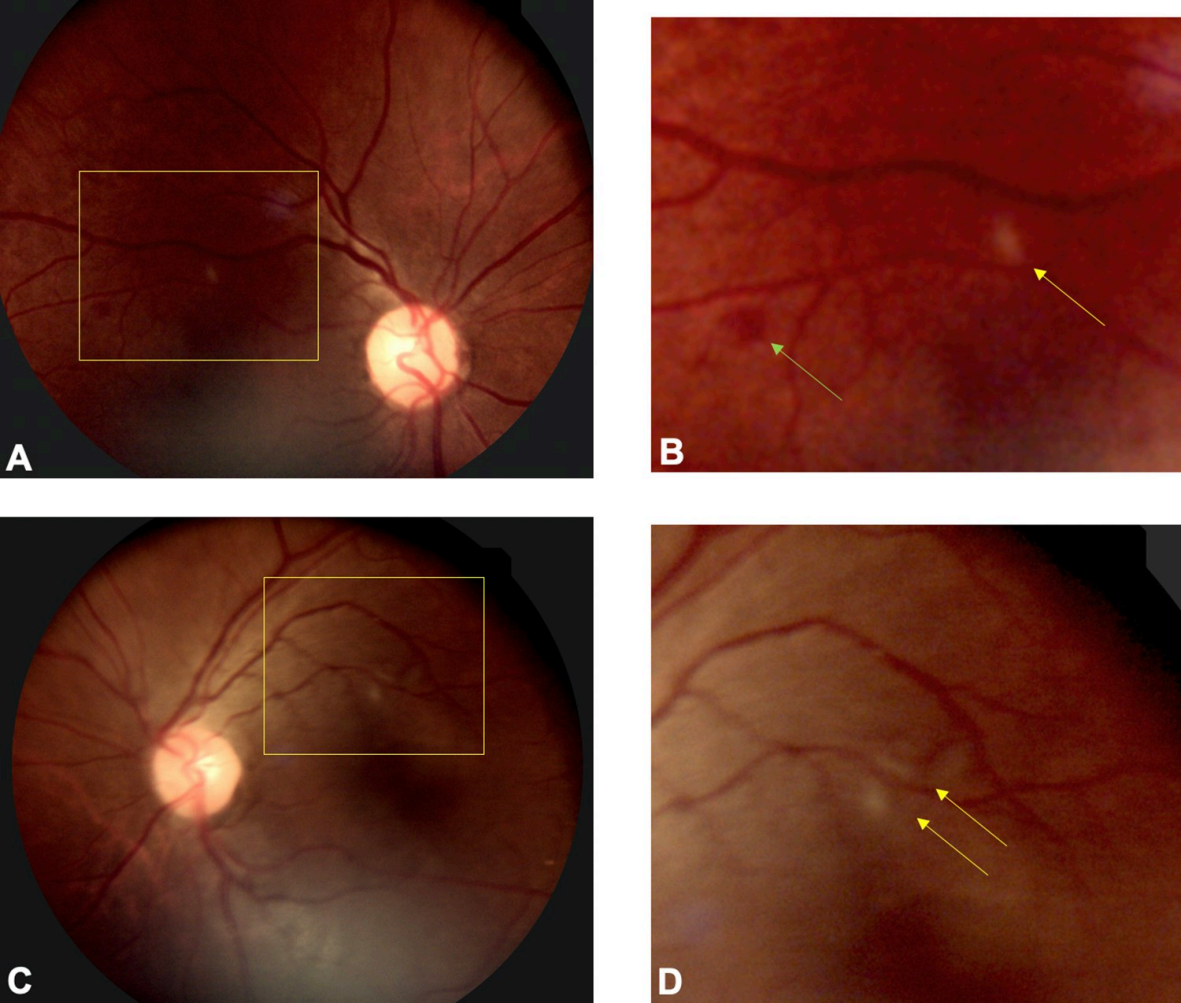
Fundus photographs of a COVID-19 patient with subtle bilateral retinopathy. (A) Fundus photo of the right eye (A) shows cotton wool spots and retinal hemorrhage (yellow inset). (B) A higher magnification view of the inset shows peri-arterial cotton wool spot (yellow arrow) and dot blot hemorrhage green. (C). Fundus photo of the left eye shows focal peri-arterial cotton wool spots (inset). (D) A higher magnification view of the cropped inset shows two cotton wool spots adjacent to retinal arteriole.

Twelve of the 60 patients (20%) who underwent analyses showed evidence of retinopathy that met case definition criteria for COVID-19 associated retinopathy and were included in statistical analyses (Figs 1 and 2). Other types of retinopathies that were observed, which were excluded from COVID-19 retinopathy analyses, including diabetic retinopathy in 3 patients (18%), hypertensive retinopathy in 1 patient (2%) and 2 classified as other types of retinopathy (12%, S1 Table).

Other ocular findings included conjunctival chemosis in 6 (10%), conjunctivitis in 2 (3%), conjunctival injection in 1 patient (2%), and subconjunctival hemorrhage in 1 (2%) patient. Cataract was also documented in 24 patients (40%, S2 Table).

### Factors associated with COVID-19 retinopathy

We further assessed whether patient demographic or baseline health factors, COVID-19 NIH severity scale, laboratory parameters or hospital interventions were associated with COVID-19 retinopathy. The median age and interquartile range (Q1, Q3) with COVID-19 retinopathy was 51.5 years (41, 56) and significantly lower than individuals without retinopathy at 62.5 years (48.5, 72.5) with an unadjusted odds ratio of 0.75 (95% CI 0.58–0.95, p = 0.01). Sex was not associated with a greater risk of retinopathy (p>0.05, Table 1, S1 and S2 Datas).

**Fig 3:**
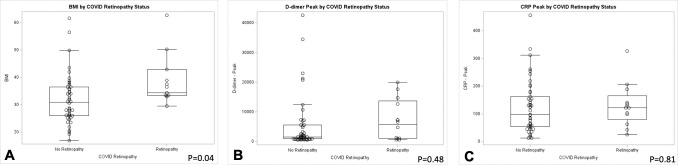
Box-and-whisker plots compare factors associated with retinopathy. (A) Median body mass index was greater in individuals with retinopathy than those without retinopathy (p = 0.04). (B) Peak D-dimers were numerically increased in COVID-19 retinopathy patients compared to those without retinopathy, but this comparison was not statistically significant (p = 0.48). (C) Median C-reactive protein exceeded normal values in both groups but were not significantly different when comparing patients with and without retinopathy (p = 0.81).

The majority of patients with COVID-19 retinopathy were black race, and the odds of COVID-19 retinopathy in Black patients was 2.27 (95% CI 0.44–11.67, p = 0.18) the odds among other patients. Patients with COVID-19 retinopathy also tended to have a higher BMI than those without. Specifically, the median BMI in patients with COVID-19 retinopathy was 34.3 (33.3, 42.8) compared to 30.9 (26.0, 36.5) in patients without COVID-19 retinopathy (p = 0.04, Fig 3). Moreover, the odds of having a BMI greater than 30 was 9.2 times higher in patients with COVID-19 retinopathy (OR 9.2 for BMI ≥ 30, 95% CI 1.1–77.6).

The odds of retinopathy were 4.53 (95% CI 0.53–38.48, p = 0.19) higher in patients with critical illness than in patients without critical illness. However, most patients were classified as critical in this cohort according to NIH severity grading given their hospitalization for respiratory failure with or without ICU admission. Besides obesity, none of the other baseline comorbidities were significantly associated with the prevalence of COVID-19 retinopathy.

Laboratory parameters were assessed to determine potential relationships between inflammation and coagulation pathways and retinopathy (Table 2). Patients with retinopathy showed a higher median peak D-dimer (Q1, Q3) of 5703 ng/mL (1018, 13746) than those patients without retinopathy with a peak D-dimer of 1549 ng/mL (889, 5595), but were not statistically significant (p = 0.48). Interestingly, the average hematocrit nadir was lower in patients with retinopathy at 29.0% (25.7, 36.0) than in individuals without retinopathy at 34.3% (28.8, 38.1). The mean peak platelets count was 410.5 x 10^9^ cells/L (365.5, 499.5) in patients with COVID-19 retinopathy than in those without retinopathy at 336 x 10^9^ cells/L (280, 438.5, p = 0.10). No significant differences were observed across other hematologic parameters including white blood cell counts or lymphocyte counts (i.e., peaks and nadirs for all values).

**Table 2:** Laboratory findings and medications in COVID-19 subjects*.

Laboratory Values	All (n = 60 patients)	COVID-19 Retinopathy (n = 12 patients)	No COVID-19 Retinopathy (n = 48 patients)	Odds Ratio	p-value
**D-dimer, Peak, mg/mL**	1583 (906, 7032)	5703 (1018, 13746)	1548.5 (889, 5595)	1.03 (0.96, 1.10)^1^	0.48
**Fibrinogen, Nadir, mg/dL**	363 (211, 488) (n = 25)	488 (175, 589)	353 (266, 488)	1.07 (0.66, 1.74)^2^	0.83
**C-reactive protein, mg/L**	111.4 (57.1, 162.9) (n = 57)	121.7 (80.1, 165.1)	97.6 (53.7, 162.9)	1.04 (0.74, 1.46)^3^	0.81
**Interleukin-6, pg/mL**	5.8 (2.3, 9.2) (n = 9)	2 (2, 25)	6.5 (3.7, 9.2)	n/a	0.75
**Platelets, Peak, x10**^**9**^ **cells/L**	348 (292, 457.5)	410.5 (365.5, 499.5)	336 (280, 438.5)	1.72 (0.99, 2.99)^2^	0.10
**Platelets, Nadir, x10**^**9**^ **cells/L**	211 (150.5, 248.5)	218 (151.5, 259)	210 (150.5, 244.5)	1.19 (0.51, 2.81)^2^	0.73
**Hematocrit, Nadir, %**	33.4 (27.6, 37.5)	29.0 (25.7, 36.0)	34.3 (28.8, 38.1)	0.66 (0.39, 1.14)^4^	0.13
**WBC, Peak, x10**^**9**^ **cells/L**	13.3 (7.4, 16.9)	14.8 (11.8, 18.3)	12 (7.15, 16.1)	1.23 (0.82, 1.85)^4^	0.33
**WBC, Nadir, x10**^**9**^ **cells/L**	6.1 (4.2, 8.0)	5.9 (4.1, 8.4)	6.2 (4.4, 8.0)	0.84 (0.26, 2.74)^4^	0.78
**Lymphocytes, Peak, x10**^**9**^ **cells/L**	16 (8, 30), (n = 51)	16 (9, 30)	16 (9, 30)	1.02 (0.79, 1.33)^4^	0.87
**Lymphocytes, Nadir, x10**^**9**^ **cells/L**	7 (3, 13), (n = 47)	7 (3, 14)	7 (3, 14)	0.92 (0.60, 1.43)^4^	0.72

*Values represented as medians and interquartile ranges (Q1, Q3)

^1^Per 1000 unit increase

^2^Per 100 unit increase

^3^Per 50 unit increase

^4^Per 5 unit increase

Interventions assessed included mechanical ventilation, vasopressor requirement, continuous renal replacement therapy, ECMO, anticoagulation (beyond low molecular weight heparin), and corticosteroid use (Table 3). Although the numbers of patients requiring extracorporeal membrane oxygenation (ECMO) was limited (n = 8), the odds of ECMO were higher among patients with COVID-19 retinopathy than in those without (OR = 5.50, 95% CI 1.14–26.63, p = 0.04). The odds of anticoagulation were also higher among patients with COVID-19 retinopathy than those without (OR = 3.27, 95% CI 0.65–16.63, p = 0.19).

**Table 3:** Interventions in COVID-19 subjects with and without retinopathy.

Interventions	All (n = 60)	COVID-19 Retinopathy (n = 12)	No COVID-19 Retinopathy (n = 48)	Odds Ratio (95% CI)	p-value
Mechanical Vent	17 (28%)	5 (42%)	12 (25%)	3.33 (0.55, 20.22)	0.23
Vasopressors	4 (7%)	2 (17%)	2 (4%)	4.60 (0.58, 36.67)	0.17
Continuous renal replacement therapy	2 (3%)	1 (8%)	1 (2%)	4.27 (0.25, 73.75)	0.36
Extracorporeal Membrane O2 (ECMO)	8 (13%)	4 (33%)	4 (8%)	5.50 (1.14, 26.63)	0.04
Lovenox	27 (45%)	3 (25%)	24 (50%)	0.33 (0.08, 1.38)	0.20
Anticoagulation beyond Lovenox	39 (65%)	10 (83%)	29 (48%)	3.27 (0.65, 16.63)	0.19
Corticosteroid	25 (42%)	6 (50%)	19 (40%)	1.52 (0.43, 5.44)	0.53

### Multivariate logistic regression modeling

Based on the observed univariate associations, we assessed the contribution of age, BMI, hematocrit nadir and anticoagulation to retinopathy (Table 4). Adjusted estimates showed that odds of retinopathy were higher with younger age (aOR 0.95, 95% CI 0.90–1.01, p = 0.095), increased BMI (aOR 1.08, 95% CI 1.00–1.18, p = 0.056), anticoagulation (aOR 3.94, 95% CI 0.61–25.6, p = 0.15) and hematocrit nadir (aOR 0.91, 95% CI 0.79–1.05, p = 0.19). However, these findings were not statistically significant.

**Table 4:** Multivariable model results.

	Adjusted Odds Ratio (95% CI)	p-value
**Model 1**		
**Age, Years**	0.95 (0.90, 1.01)	0.095
**Body mass index**	1.08 (1.00, 1.18)	0.056
**Anticoagulation**	3.94 (0.61, 25.60)	0.152
**Hematocrit, Nadir, %**	0.91 (0.79, 1.05)	0.189

### SARS-CoV-2 RT-PCR

We also assessed the tear film for SARS-CoV-2 RNA by RT-PCR. Nineteen of 120 samples (16%, 95% CI 0.98–0.24) tested positive for SARS-CoV-2 RT-PCR with either one or two SARS-CoV-2 targets. Fifteen of 60 patients (25%, 95% CI 0.15–0.38) showed positive RT-PCR tests in either eye with 4 of 60 patients (6.7%, 95% 0.019–0.16) testing positive in both.

Five eyes of 4 patients were positive with all three targets (N2, E, and RNAse P genes). Thirteen eyes of 10 patients were positive with the N2 gene detection and only one eye showed E gene detection. Of note, alpha variant (lineage B.117) was detected in three eyes of two patients with N2 Ct values varying between 25.9 and 31.8.

We further assessed the relationship between SARS-CoV-2 RNA positivity in the tear film with disease related factors. Patients with SARS-CoV-2 RT-PCR positive tear film showed a numerically greater duration of symptomatic COVID-19 with a mean ± SD of 11.4 ± 14.3 days of illness compared with 8.9 ± 8.2 days for those who were RT-PCR negative (p = 0.41). The number of days of hospitalization was also greater at 5.3 ± 13.6 days in individuals who were SARS-CoV-2 positive compared to 2.2 ± 6.1 days patients who tested negative (p = 0.22), but these differences were not statistically significant. No correlation was observed between SARS-CoV-2 RNA detection on the ocular surface and the presence or absence of COVID-19 retinopathy (p>0.05). Further, no differences were observed in the proportion of individuals who were SARS-CoV-2 RNA positive in tear film when stratified by NIH COVID-19 severity (i.e., critical vs. non-critical illness, p>0.05).

## Discussion

In this prospectively enrolled, cross-sectional study of hospitalized COVID-19 patients, 20% of patients showed evidence of COVID-19 retinopathy, as defined by acute signs of retinal vascular damage (i.e., cotton wool spots, retinal hemorrhage), not attributable to diabetes, hypertension or other preexisting systemic vascular conditions. The prevalence of retinopathy was associated with younger age, increased BMI, and ECMO requirement in unadjusted analyses. Other factors of interest, which showed a non-statistically significant association with retinopathy included black race and NIH COVID-19 severity score.

The reported prevalence of retinopathy in COVID-19 patients has varied from 22% to 38% with higher prevalence of retinopathy in COVID-19 patients with worse severity [4, 9–17]. Factors previously associated with retinopathy include higher sequential organ failure assessment scores, respiratory failure requiring mechanical ventilation, hypotension requiring vasopressors and elevated D-dimers [4]. While D-dimers did not differ between COVID-19 patients with and without retinopathy in this COVID-19 population, wide variation and abnormally elevated D-dimers were observed in the cohort of severe COVID-19 patients. Anticoagulation, defined by medication requirements in addition to low molecular weight heparin, was also increased in patients with COVID-19 retinopathy. Coagulation diathesis may potentially be involved in the pathogenesis of retinal microvascular disease and warrants further investigation.

BMI was one particular risk factor of note that differed significantly between patients with and without retinopathy. Specifically, while the median BMI of 32.9 in the entire cohort met criteria for obesity, those with retinopathy showed a BMI of 34.3 compared with 30.9 in individuals without retinopathy. Moreover, the odds of obesity were 9 times higher in patients with retinopathy than in those without. While BMI and obesity are associated with diabetes and hypertension that may also be associated with retinopathy independent of COVID-19, the relationship of BMI and retinopathy in the setting of COVID-19 raises the question of whether components of metabolic syndrome (e.g., obesity, dyslipidemia) may contribute to endothelial dysfunction and inflammation [32, 33], which may also contribute to retinopathy. Investigations studying the precise relationships between obesity and microvascular disease are currently underway, particularly related to cardiac and thromboembolic disease [34].

In addition to our analyses of risk factors related to retinopathy, we also assessed SARS-CoV-2 RNA with RT-PCR of conjunctival tear film swabs and showed that approximately 25% of patients had positive results with a validated assay for SARS-CoV-2 detection. Interestingly, the alpha variant was also identifiable via a conjunctival swab, indicating that these samples can also be used for typing SARS-CoV-2 variants. Whether other specific variants may be detected on the ocular surface, including the more recent omicron variants, remains unknown. A clear relationship between SARS-CoV-2 RNA detection and days of COVID-19 symptomatic illness or hospitalization was not detected. The prevalence of tear film SARS-CoV-2 RNA has been estimated as high as 57% in prior studies [24], but the initial SARS-CoV-2 viral load, tear film clearance dynamics, and assay sensitivity may impact the variable prevalence between studies.

Moreover, while no clear relationships were identified between either anterior or posterior segment ocular findings and surface and tear film SARS-CoV-2 RNA, we were able to detect SARS-CoV-2 on the ocular surface of patients without conjunctival signs indicating the potential for viral RNA in the absence of ocular symptoms (i.e., asymptomatic ocular surface carrier). Detection of SARS-CoV-2 RNA may represent aerosolized virus from respiratory secretions or contiguous spread of SARS-CoV-2 within the respiratory / ocular surface mucosa. The Centers for Disease Control and Prevention currently recommends eye protection during the evaluation of patients with confirmed or suspected COVID-19, as well as in high transmission areas. Infection prevention and control practices to prevent disease transmission to health care providers, particularly eye care professionals, via the ocular surface mucosa remains paramount [25]. Whether the ocular surface is an efficient route of disease transmission is unknown, but recent studies have described ACE2 receptor and serine protease TMPRSS2 within both corneal and conjunctival epithelial cells [28, 29].

Limitations of this study include the relatively sample size and potential for selection bias given the tertiary referral center-based recruitment, which may be biased towards greater disease severity and a higher prevalence of retinopathy than other COVID-19 patient populations, including individuals with mild disease or asymptomatic infection. Interestingly, we previously described a nearly 40% prevalence of retinopathy in a retrospective study of COVID-19 patients who were hospitalized primarily in the intensive care unit setting [4]. It is plausible that increasing microvascular disease including retinopathy may be observed with higher degrees of systemic morbidity in COVID-19 patients. Moreover, while we classified patients into hypertensive or diabetic retinopathy in patients if they showed retinal findings characteristic of these diseases (e.g., exudates in diabetic macular edema), there is the possibility that these conditions could also be involved in retinopathy associated with COVID-19. While increased BMI was positively associated with retinopathy, it is possible that patients with obesity or increased BMI may have had retinopathy as well. However, univariate analyses assessing the potential increased risk of retinopathy with diabetes and hypertension did not show a significant increase in retinopathy in our series, although the comparisons were limited by their sample size. Given that ECMO has previously been associated with retinopathy [35], this intervention could also be a contributing factor. A larger cohort with an age- and gender-matched control population could better disentangle systemic comorbidities that could contribute to retinopathy, as well as interventions that could be associated with retinal findings.

Our findings from univariate analyses that age and BMI were associated with a higher rate of retinopathy warrant further study given the potential mechanisms that may be involved in the development of retinal microvascular damage. Specifically, whether elevated BMI may contribute to retinal microvascular disease pathogenesis in the setting of COVID-19 via inflammation, endothelial dysfunction or other mechanisms is unknown. Moreover, whether retinopathy may serve as a biomarker for concurrent systemic disease morbidity, coagulation diathesis, inflammation or hypoxemia also requires further understanding. Given the ongoing COVID-19 pandemic, further investigation of SARS-CoV-2 mucosal persistence, ocular surface immunity, and clinical disease findings will be needed, particularly as variants with unique transmission potential and disease features arise.

## Supporting information

S1 Table Retinopathy classification in enrolled patients. (DOCX)

S2 Table Ocular findings in COVID-19 subjects by patients and by eyes. (DOCX)

S1 Data (SAS7BDAT)

S2 Data (SAS)
