# Supplementary material for: COVID-19 and the eye: Systemic and laboratory risk factors for retinopathy and detection of tear film SARS-CoV-2 RNA with a triplex RT-PCR assay
Source: PLoS One. 2022 Nov 9;17(11):e0277301. doi: 10.1371/journal.pone.0277301 (PMC9645628; doi:10.1371/journal.pone.0277301)
Supplement: S1 Table — (DOCX) [file pone.0277301.s001.docx]

**Supplemental Table 1. Retinopathy Classification in Enrolled Patients**

|  | **Patients** | **Bilateral Patients** | **Unilateral Patients** | **Eyes** |
| --- | --- | --- | --- | --- |
| **Retinopathy** | **17** | **6** | **11** | **23** |
| **COVID-19 Retinopathy** | 12 | 2 | 10^a^ | 14 |
| **Diabetic Retinopathy** | 3 | 1 | 2 | 4 |
| **Hypertensive Retinopathy** | 1 | 1 | 0 | 2 |
| **Other Retinopathy** | 2 | 1 | 1 | 3 |

^a^One person had bilateral retinopathy, but unilateral covid retinopathy (OS COVID retinopathy, OD diabetic retinopathy)

Note: One patient is counted in both COVID retinopathy and diabetic retinopathy categories.

Bilateral patient has one eye with COVID retionapthy and one eye with diabetic retinopathy.
