# Supplementary material for: COVID-19 and the eye: Systemic and laboratory risk factors for retinopathy and detection of tear film SARS-CoV-2 RNA with a triplex RT-PCR assay
Source: PLoS One. 2022 Nov 9;17(11):e0277301. doi: 10.1371/journal.pone.0277301 (PMC9645628; doi:10.1371/journal.pone.0277301)
Supplement: S2 Table — (DOCX) [file pone.0277301.s002.docx]

**Supplemental Table 2. Ocular Findings in COVID-19 Subjects by Patients and by Eyes**

| **Ocular Findings** | **Patients (%)**  **(n=60)** | **Eyes (%)**  **(n=120)** |
| --- | --- | --- |
| Conjunctival injection | 1 (2%) | 2 (2%) |
| Conjunctivitis | 2 (3%) | 4 (3%) |
| Conjunctival chemosis | 6 (10%) | 11 (9%) |
| Subconjunctival hemorrhage | 1 (2%) | 1 (1%) |
| Cataract | 24 (40%) | 48 (40%) |
